# Supplementary material for: MG-Pe: A Novel Galectin-3 Ligand with Antimelanoma Properties and Adjuvant Effects to Dacarbazine
Source: Int J Mol Sci. 2022 Jul 11;23(14):7635. doi: 10.3390/ijms23147635 (PMC9317553; doi:10.3390/ijms23147635)
Supplement: Supplementary file 1 [file ijms-23-07635-s001.zip › ijms-1775439-supplementary.pdf]

Supplementary Materials:

Table S1. Galectin inhibitors, effects and clinical trials.

Molecule	Galectin inhibitors	Effect	Clinical trials
Davanat (GM-CT-01) [49][39]	Gal-1	Antitumor – solid tumors	phase 1 (NCT00054977)
Anginex (β-pep25) [81]	Gal-1	Antiangiogenic and antitumour effects	-
DiBenzoFulvene (DBF) [82]	Gal-1	Angiostatic activity	-
OTX008 (PTX008/Calixarene 0118) [83,84]	Gal-1	Antitumor and antiangiogenic activity	-
GR-MD-02 (Belapectin) [85]	Gal-3	NASH (Nonalcoholic Steatohepatitis) cirrhosis	phase 3 (NCT04365868)
Modified Citrus Pectin (MCP, PectaSol-C) [86]	Gal-3	Urinary bladder cancer (UBC)	-
GB0139 (TD139) [87,88]	Gal-3	Idiopathic Pulmonary Fibrosis<br><br>COVID-19	phase 2b (NCT03832946)<br><br>Phase 2 (NCT04473053)
GB1211 [89,90]	Gal-3	Liver fibrosis	Phase2a trial (NCT03809052)
MBG453 [91,92]	Gal-3	Myelodysplastic Syndrome or Chronic Myelomonocytic Leukemia-2<br><br>Acute Myeloid Leukemia (AML)	Phase 3 clinical trials in Combination with Azacitidine (NCT04266301)<br><br>Phase 2 clinical trials in combination with Azacitidine and Venetoclax (NCT04150029)

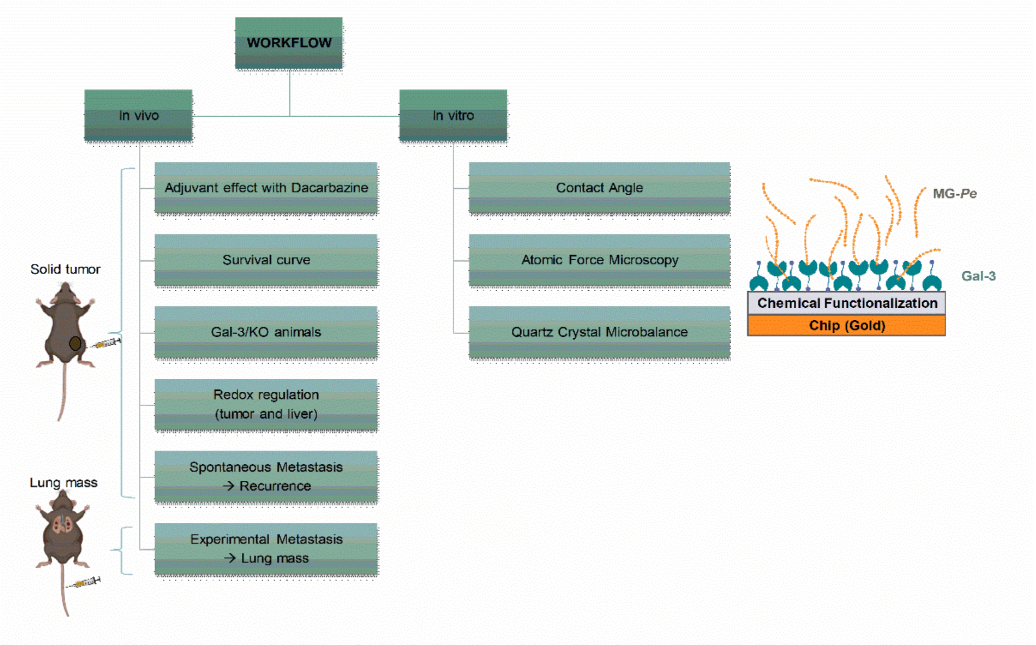

Figure S1. A workflow of the techniques described in the material and methods.

Western Blotting

Pellet from B16-F10 and SK-MEL-28 cells was lysed in a buffer containing 50 mM Tris, pH 7.5, 150 mM NaCl, 10 mM MgCl2,0.5 mM DTT, 1 mM EDTA, 10% glycerol, 2% SDS, 1% Triton X-100 and protease inhibitors (aprotinin 2µg/mL and PMSF 1mM) for 30 min on ice. After centrifugation (13.000 *rpm* for 10 min at 4°C), the protein supernatants were collected, quantified by the Bradford method (Bio-Rad, Richmond, CA) and 30µg total proteins were separated by SDS-PAGE. Next, the proteins were transferred onto PVDF membranes, blocked with 5% non-fat dry milk for 1 h and incubated overnight with primary antibodies: M3/38 rat anti-Gal-3 Hybridoma (1:200), and Tubulin 1:1000, Calbiochem/Merck, USA, (DM1A). The secondary antibodies are conjugated to HPRO. The samples were visualized with the chemiluminescent substrate ECL (GE Healthcare). B16-F10 in culture does not express Gal-3, but it is possible that, when subjected to *in vivo* environment, the cells resume its expression, which was shown for other systems. For example, Machado and colleagues (2014) showed in the tumor parenchyma, during tumor angiogenesis, that Gal-3 can be restored in melanoma cell lines, which previously did not express this protein [59].

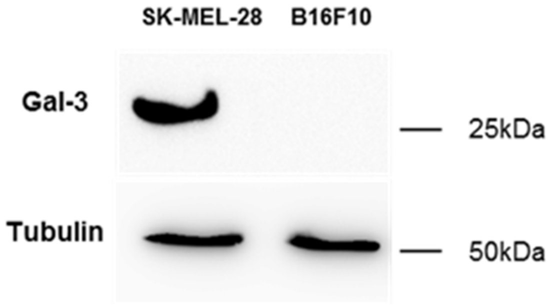

Figure S2. Western Blotting assay demonstrates the absence of Gal-3 in B16-F10 cells at the time of inoculation of these cells in mice, using as controls the SK-MEL-28 cell that has the presence of galectin and tubulin.
